# Supplementary material for: Hybrid optimization technique for matrix chain multiplication using Strassen’s algorithm
Source: F1000Res. 2025 May 27;14:341. Originally published 2025 Mar 27. [Version 2] doi: 10.12688/f1000research.162848.2 (PMC12000801; doi:10.12688/f1000research.162848.2)
Supplement: Peer review history

# Reviewer report: Reviewer response for version 2

**DOI:** 10.5256/f1000research.180873.r387461

**Published:** 2025-06-03

**Authors:** Tekle Gemechu Dinka

**Affiliations:** 1 Applied Mathematics, Adama Science and Technology University, Adama, Oromia, Ethiopia

Dear Authors, the work was well done. But, let you consider the following few comments, for indexing.

 1. In Methods Section, see typo error,  O(n 2.81)!

 2. In Fig1, are you sure why the values of all absolute errors are in range [0, 1] ?

  Is that relative absolute error or just absolute error? Check it, please!

 3. Based on the first comment 1 above, let you check the entire work roughly, once! (Optional)

 Note: Do not send it back to me again, for 3 ^rd^ time revision!

 Suggestion: Accepted for publication (Approved).

 Thanks!

Is the work clearly and accurately presented and does it cite the current literature?

Yes

If applicable, is the statistical analysis and its interpretation appropriate?

Not applicable

Are all the source data underlying the results available to ensure full reproducibility?

Partly

Is the study design appropriate and is the work technically sound?

Yes

Are the conclusions drawn adequately supported by the results?

Yes

Are sufficient details of methods and analysis provided to allow replication by others?

Yes

Reviewer Expertise:

Applied Mathematics

I confirm that I have read this submission and believe that I have an appropriate level of expertise to confirm that it is of an acceptable scientific standard.

# Reviewer report: Reviewer response for version 1

**DOI:** 10.5256/f1000research.179110.r377063

**Published:** 2025-04-18

**Authors:** Bharati Ainapure

**Affiliations:** 1 Vishwakarma University, Pune, Maharashtra, India

**Hybrid optimization technique for matrix chain multiplication using Strassen’s algorithm**

 Srinivasarao Thota, Thulasi Bikku, Rakshitha T

 The manuscript presents a novel and practical hybrid optimization framework that combines Dynamic Programming-based Matrix Chain Multiplication (MCM) with Strassen’s Algorithm. The paper demonstrates significant contributions in terms of computational efficiency, reduced memory usage, and real-world applicability. The authors have implemented the hybrid model with robust experimental validation and well-documented algorithms.

**Review comments: **

1. The paper introduces a hybrid optimization framework that smartly integrates MCM with Strassen’s Algorithm. This approach is novel and significantly improves computational performance while maintaining numerical integrity for large matrices.
2. The authors clearly articulate the limitations of traditional MCM and Strassen’s algorithm when used individually, thus justifying the need for a hybrid approach.
3. The pseudocode for **matrix_chain_order**, **strassen_matrix_multiply**, and the hybrid multiplication routine is clearly written, logically structured, and reproducible.
4. The inclusion of performance benchmarks (execution time, memory usage, scalar operations, accuracy) across various matrix sizes provides compelling evidence of the hybrid method's benefits.
5. The detailed step-by-step numerical example enhances understanding and demonstrates the practical application of the hybrid algorithm.
6. The figures (execution time, speedup, scalar multiplication, memory usage, correlation heatmap) visually substantiate the claims and provide insights into the hybrid model’s behavior across scenarios.
7. The selective application of Strassen’s method to matrices with dimensions ≥ 128x128 is a thoughtful design decision that avoids the recursive overhead on smaller matrices.
8. The authors provide links to GitHub and Zenodo for full access to the code and data, promoting transparency and reproducibility in computational research.
9. The background section and references demonstrate familiarity with both foundational and current research trends in matrix multiplication, including modern alternatives to Strassen’s algorithm.
10. The conclusion aptly summarizes the findings and points toward promising future directions such as parallelization and adaptive thresholding for broader applicability.

Is the work clearly and accurately presented and does it cite the current literature?

Yes

If applicable, is the statistical analysis and its interpretation appropriate?

Yes

Are all the source data underlying the results available to ensure full reproducibility?

Yes

Is the study design appropriate and is the work technically sound?

Yes

Are the conclusions drawn adequately supported by the results?

Yes

Are sufficient details of methods and analysis provided to allow replication by others?

Yes

Reviewer Expertise:

Algorithms

I confirm that I have read this submission and believe that I have an appropriate level of expertise to confirm that it is of an acceptable scientific standard.

# Response

**Published:** 2025-04-20

**Authors:** Rakshitha T

**Affiliations:** Mathematics, Eritrea Institute of Technology, Mai Nefhi, Mai Nefhi, Eritrea

Thank you for your thoughtful review and kind feedback on our manuscript. We truly appreciate your encouraging remarks and are grateful for your approval. Your insights and support have been incredibly motivating and have significantly contributed to improving the quality of our work.

# Reviewer report: Reviewer response for version 1

**DOI:** 10.5256/f1000research.179110.r377065

**Published:** 2025-04-15

**Authors:** Rajak Shaik

**Affiliations:** 1 SRM University AP, Mangalagiri, Andhra Pradesh, India

**Summary of the Work: **The manuscript presents a hybrid optimization strategy that combines Matrix Chain Multiplication (MCM) using dynamic programming with Strassen’s Algorithm for selective acceleration of large matrix multiplications. The approach addresses computational inefficiencies inherent in traditional MCM and standalone Strassen’s methods. The hybrid technique is validated through a combination of theoretical analysis, pseudocode implementation, performance benchmarking, and a clear numerical example. The paper is relevant to fields including scientific computing, deep learning, and high-performance matrix operations.

  

1. **Innovation**: The integration of Strassen’s algorithm into MCM is a smart optimization strategy that yields tangible performance gains.
2. **Clarity of Methodology**: The algorithm is clearly described with detailed pseudocode and complexity analysis, making the methodology reproducible.
3. **Experimental Rigor**: The authors conduct comprehensive benchmarks covering execution time, memory usage, and numerical accuracy. Comparative plots strengthen the conclusions.
4. **Balanced Hybridization**: The selective application of Strassen’s algorithm based on matrix size (≥128) is well-justified and effectively balances performance and overhead.
5. **Applicability**: The work is applicable across several domains such as graphics, neural network training, and physics simulations—making it broadly relevant.
6. **Reproducibility**: The codebase and data are made publicly available through GitHub and Zenodo, aligning with modern best practices in open science.
7. **Well-Structured Paper**: The manuscript is logically organized, guiding the reader smoothly from problem motivation to algorithm design and results.
8. **Numerical Example**: The inclusion of a worked-out example with intermediate computations illustrates the real-world practicality of the proposed technique.
9. **Graphical Comparisons**: Figures such as execution time charts, memory comparisons, and a correlation heatmap visually reinforce the paper’s findings.
10. **Future Scope**: The conclusion thoughtfully outlines future directions including parallelization and adaptive thresholds, highlighting the research’s extensibility.

**Minor Suggestions (Optional Enhancements for a Revised Version)**

 While I recommend acceptance as-is, the authors may consider the following enhancements in future iterations:

- Elaborate on the **numerical stability** trade-offs when using Strassen’s Algorithm in floating-point computations.
- Explore **adaptive thresholding mechanisms** in more detail, possibly as an appendix or supplementary material.
- Include a brief comparison with more recent fast matrix multiplication techniques like Coppersmith-Winograd or Karatsuba-like methods in the background.

**Final Recommendation:**

 The manuscript makes a valuable contribution to computational optimization in matrix algebra. It is well-written, methodologically sound, and thoroughly validated.

**I recommend this paper for acceptance without revisions.**

Is the work clearly and accurately presented and does it cite the current literature?

Yes

If applicable, is the statistical analysis and its interpretation appropriate?

Yes

Are all the source data underlying the results available to ensure full reproducibility?

Yes

Is the study design appropriate and is the work technically sound?

Yes

Are the conclusions drawn adequately supported by the results?

Yes

Are sufficient details of methods and analysis provided to allow replication by others?

Yes

Reviewer Expertise:

AI & ML

I confirm that I have read this submission and believe that I have an appropriate level of expertise to confirm that it is of an acceptable scientific standard.

# Response

**Published:** 2025-04-17

**Authors:** Rakshitha T

**Affiliations:** Mathematics, Eritrea Institute of Technology, Mai Nefhi, Mai Nefhi, Eritrea

Thank you for your thoughtful review and positive feedback on our manuscript. We sincerely appreciate your encouraging comments and are grateful for your approval. Your insights and support have been highly motivating and have contributed to enhancing the quality of our work.

# Reviewer report: Reviewer response for version 1

**DOI:** 10.5256/f1000research.179110.r377061

**Published:** 2025-04-15

**Authors:** Kranthi Kumar Singamaneni

**Affiliations:** 1 Symbiosis Institute of Technology, Hyderabad, India

**Title:** Hybrid optimization technique for matrix chain multiplication using Strassen’s algorithm

**Authors:** Srinivasarao Thota, Thulasi Bikku, Rakshitha T

 This article is a well-structured and methodologically sound study that integrates Strassen’s Algorithm into the traditional Matrix Chain Multiplication (MCM) optimization framework.

**Comments:**

1. **Clear Motivation & Problem Statement:**
  - Addresses computational inefficiencies in standard MCM (O(n³) time).
  - Identifies Strassen’s Algorithm (O(n^2.81)) as a viable method to accelerate large matrix multiplications.
2. **Innovative Hybrid Approach:**
  - Combines Dynamic Programming (DP) for optimal matrix order selection.
  - Applies Strassen’s Algorithm conditionally for matrix dimensions ≥128×128 to avoid unnecessary overhead.
  - Introduces memory-efficient techniques using rolling DP arrays.
3. **Algorithm & Pseudocode:**
  - Well-structured Python code for MCM-DP and Strassen’s recursive multiplication.
  - Implements hybrid decision-making for performance tuning.
  - Ready-to-run and reproducible with publicly available code (GitHub, Zenodo).
4. **Use of Numerical Example:**
  - Provides a complete walkthrough with four sample matrices.
  - Demonstrates cost calculations, order of operations, and execution trace.
5. **Data Transparency & Open Science:**
  - Source code available under GPL-3.0 license on GitHub.
  - Dataset and figures provided via Zenodo.
  - Heatmaps and comparative charts (Figures 1–8) provide strong visual insights.

This paper introduces a **practical and efficient hybrid technique** for matrix chain multiplication that is **well-supported theoretically and empirically**. It is especially suitable for **large-scale, performance-sensitive computing domains** such as scientific simulations and deep learning.

 The paper is complete, methodically presented, and ready for getting indexed in bibliographic databases.

Is the work clearly and accurately presented and does it cite the current literature?

Yes

If applicable, is the statistical analysis and its interpretation appropriate?

Partly

Are all the source data underlying the results available to ensure full reproducibility?

Yes

Is the study design appropriate and is the work technically sound?

Yes

Are the conclusions drawn adequately supported by the results?

Yes

Are sufficient details of methods and analysis provided to allow replication by others?

Yes

Reviewer Expertise:

Quantum Computing, Cyber Security, Data Analytics

I confirm that I have read this submission and believe that I have an appropriate level of expertise to confirm that it is of an acceptable scientific standard.

## References

[1] : Hybrid optimization technique for matrix chain multiplication using Strassen’s algorithm. *F1000Research* .2025;14: 10.12688/f1000research.162848.1 10.12688/f1000research.162848.1 PMC1200080140242469

# Reviewer report: Reviewer response for version 1

**DOI:** 10.5256/f1000research.179110.r374494

**Published:** 2025-04-15

**Authors:** Tekle Gemechu Dinka

**Affiliations:** 1 Applied Mathematics, Adama Science and Technology University, Adama, Oromia, Ethiopia

**Review Report**

 Title: **Hybrid optimization technique for matrix chain multiplication using Strassen’s algorithm**

**Authors:** Srinivasarao Thota, Thulasi Bikku, Rakshitha T

 Recommendation: **ACCEPTED**, with Revisions Required! (Approved With Reservation)

 1) General Comment: Dear authors, your work seems so interesting, well done! The background (abstract), the methods, results and conclusions parts all sound good. But, please check the clarity and or achievement of overall works (all parts, results in tables & graphs), check journal styles, use of references….before publication! Also, consider all associated comments! Check all mathematical equations in the paper!

 2) It seems your introduction is not so detail. To increase readability or quality of your work, you may say more on the past works and current researches focusing on the MCM technique and or Strassen’s algorithms (including improvements or drawbacks), if any (Optional!). You can consider some of listed references below!

 3) Please use equation numbering for the equations, in your paper.

 4) What are i & j in cost equation on page 3?

 5) The section on “Strassen’s algorithm for matrix multiplication” on page 4, needs corrections. In the four divisions of matrices A & B , the first division of first equation has to be rewritten (check the first equation to give corrections!).



 In the Strassen’s algorithm, also there should be included, the product matrix C, such that C = AB, and 

 

 Please check it all, on page 4!

 6) In combining MCM and strassen’s algorithm to get your proposed method, on page 5, where is the mathematical formulation? Otherwise, how was the code for hybrid algorithm written? Try to think over!

 7) In subsection 3.2, first sentence, rewrite as, "we provide **the time complexity analysis** of the…

 8) What is your evidence for the following (on page 6, second paragraph)?

 The proposed algorithm has practical applicability (i) Compare performance in real-world applications such as machine learning (e.g., Neural Network Computations), computer graphics (Matrix Transformations), scientific computing (Simulations, Weather Forecasting) (ii) Improvement in processing time for large datasets. You may give references.

 9) Strassen’s algorithm reduces computation cost complexity from O(n^3) to O(n^2.81). And also from eight to seven multiplications. What about your hybrid method? Where /what is the order of reduction?

 10) In figure 1, why the maximum value from vertical y-axis is 1? Or why Range = [0, 1]? Otherwise, vertical axis must represent mean relative error, not mean absolute error. Please, may you, check it!

 11) In figure 2,  hybrid  method and strassen’s algorithm are of O(n^2.81). So where is the complexity reduction? How is the improvement? Otherwise, why your method is more efficient or more accurate? Describe clearly how good memory usage of your method made it perform better.

 12) On page 9, in last paragraph, rewrite as “figure 5”  it is not figure 6.

 13) On page 10, below figure 6, rewrite as “one can observe that….

**Good Luck!**

 You may refer the following (It is good to do but Optional!)

 1. *Strassen, Volker (1969 [Ref-1]). "Gaussian Elimination is not Optimal". Numer. Math. **13** (4): 354–356.* *doi* *:* *10.1007/BF02165411* *.* *S2CID*** *121656251* *.*

 2. *Skiena, Steven S. (1998), "§8.2.3 Matrix multiplication", The Algorithm Design Manual, Berlin, New York:* *Springer-Verlag* *,* *ISBN*** *978-0-387-94860-7* .

 3. *D'Alberto, Paolo; Nicolau, Alexandru (2005).* *Using Recursion to Boost ATLAS's Performance* *(PDF). Sixth Int'l Symp. on High Performance Computing.*

 4. *Huang, Jianyu; Smith, Tyler M.; Henry, Greg M.; van de Geijn, Robert A. (13 Nov 2016).* *Strassen's Algorithm Reloaded* *.* *SC16: The International Conference for High Performance Computing, Networking, Storage and Analysis* *. IEEE Press. pp. 690–701.* *doi* *:* *10.1109/SC.2016.58* *.* *ISBN*** *9781467388153* *. Retrieved 1 Nov 2022.*

 5. *Winograd, S. (October 1971 [Ref-2]).* *"On multiplication of 2 × 2 matrices"* *. Linear Algebra and Its Applications. **4** (4): 381–388.* *doi* *:* *10.1016/0024-3795(71)90009-7* *.*

 6. *Karstadt, Elaye; Schwartz, Oded (2017-07-24).* *"Matrix Multiplication, a Little Faster"* *. Proceedings of the 29th ACM Symposium on Parallelism in Algorithms and Architectures. ACM. pp. 101–110.* *doi* *:* *10.1145/3087556.3087579* *.* *ISBN*** *978-1-4503-4593-4* *.*

Is the work clearly and accurately presented and does it cite the current literature?

Yes

If applicable, is the statistical analysis and its interpretation appropriate?

Not applicable

Are all the source data underlying the results available to ensure full reproducibility?

Partly

Is the study design appropriate and is the work technically sound?

Yes

Are the conclusions drawn adequately supported by the results?

Yes

Are sufficient details of methods and analysis provided to allow replication by others?

Yes

Reviewer Expertise:

Applied Mathematics

I confirm that I have read this submission and believe that I have an appropriate level of expertise to confirm that it is of an acceptable scientific standard, however I have significant reservations, as outlined above.

## References

[1] : Gaussian elimination is not optimal. *Numerische Mathematik* .1969;13(4) : 10.1007/BF02165411 354-356 10.1007/BF02165411

[2] : On multiplication of 2 × 2 matrices. *Linear Algebra and its Applications* .1971;4(4) : 10.1016/0024-3795(71)90009-7 381-388 10.1016/0024-3795(71)90009-7

# Response

**Published:** 2025-04-17

**Authors:** Rakshitha T

**Affiliations:** Mathematics, Eritrea Institute of Technology, Mai Nefhi, Mai Nefhi, Eritrea

1. As per the comment from the reviewer, we have revised all the mathematical equations in the paper.

 2. The introduction section is revised and includes more details in the revised version.  

 3. Equation numbers are given wherever required in the revised manuscript. 

 4. *i* is the starting index of the subchain of matrices (i.e., matrix *A _i_*), and *j* is the ending index of the subchain of matrices (i.e., matrix *A _j_*). This information is included in the revised manuscript. 

 5. Matrices are replaced with the correct notations A and C as suggested by the reviewer. 

 6. The hybrid algorithm is a process that consists of two main phases: Matrix Chain Order Optimization and Strassen’s Algorithm. These are explained in Sections 2.1 and 2.2. The hybrid algorithm is given in Section 3.

 7. As per the suggestion, we have written the sentence. 

 8. As per the comment, we have included some references and cited them in the introduction section. 

 9. The hybrid method uses matrix chain multiplication and Strassen’s algorithm to reduce the time complexity of the matrix multiplication. The hybrid algorithm contains seven multiplications instead of eight multiplications. 

 10. The vertical y-axis in Figure 1 is capped at 1 because the range [0, 1] typically represents a normalized scale, often used for relative errors or percent errors. If it shows mean absolute error, values close to or up to 1 imply that errors are nearly as large as the actual values-unrealistic in high-precision tasks. Therefore, either the plot shows mean relative error, not mean absolute error, or the y-axis is misleadingly scaled, and the label needs correction.

 11. The proposed hybrid method is more efficient because it smartly reduces total operations using MCM, and uses Strassen only when beneficial, while maintaining low memory usage-leading to real-world speedups and better numerical stability.

 12. Updated in the revised manuscript. 

 13. As per the valuable comment from the reviewer, we have updated it.
